# Nanofiltration Process for Enhanced Treatment of RO Brine Discharge

**DOI:** 10.3390/membranes11030212

**Published:** 2021-03-18

**Authors:** Mohamed E.A. Ali

**Affiliations:** Egypt Desalination Research Center of Excellence (EDRC) & Hydrogeochemistry Department, Desert Research Center, Cairo 11753, Egypt; m7983ali@gmail.com

**Keywords:** nanofiltration, brine discharge, hypothetical electrolyte, saturation indices, recycling

## Abstract

Brine discharge of reverse osmosis (RO) desalination plants represents a challenge for both inland and coastal desalination plants. Zero-liquid discharge (ZLD) can be accomplished by using additional stages of RO, which can recycle that brine water, but the key challenge is the high concentration of divalent salts. These divalent salts (especially calcium and magnesium salts) forms a scaling layer on the RO membrane surfaces and hence shorten the life-time of the membranes. In this study, the nanofiltration (NF) procedure was used to remove divalent ions from the brine discharge to minimize the load on additional stages of RO membranes. One of the most critical considerations influencing the selection of an effective NF is the water type, which is expected here by calculation of some hydrochemical parameters (major ions, hypothetical soluble salts (electrolyte), and saturation indices). NF experiments were undertaken on a lab-scale using a low-pressure hand-made system of 4–7 bar. Synthetic single salts solutions and two real brine water discharge (brackish (BWRO) and seawater (SWRO) desalination plants) were used as a feed solution for NF system. The chemical characteristics of the RO-feed, RO-brine, NF-permeate, and NF-reject in were investigated. Electrolyte concentrations and saturation indices were determined based on the concentration of the major ions and the NETPATH software package, respectively. Calculations reveal that the brine concentrate samples contained mostly MgSO_4_ and MgCl_2_ soluble salts. The results show that 79–89% of the total dissolved salts (TDS) and 96–98% of the total hardness (TH) were retained using the NF process. The salt rejection of the NF membrane follows the order of CaSO_4_, Na_2_SO_4_, MgSO_4_, MgCl_2_, and NaCl with a percent of 97.4, 97.3, 95.2, 93.4, and 79%, respectively.

## 1. Introduction

Reverse osmosis (RO) is the most effective technology for desalinating brackish and saline groundwater because it has a higher recovery when the feed water contains low salt concentrations [1]. For any RO desalination plant, there are two main primary streams: desalinated water and extremely saline wastewater (brine water). Brine (concentrate) is the by-product of the desalination process that, due to its high salinity, adversely affects the environment. In the case of inland desalination plants, this brine is mainly discharged into deep groundwater aquifers [2,3,4,5]. Since this brine solution includes high salt concentration as well as residuals of pre-treatment chemicals and the chemicals for membrane cleaning; it deteriorates the groundwater quality [6,7,8,9]. 

Brine disposal costs range from about 5 to 30% of the overall desalination costs, which means that any improvement progress or development in disposal management and treatment can be translated into a decrease in desalination costs [10]. There are many research studies have been undertaken to find for an effective and efficient application for brine disposal management [11,12,13,14,15]. To select an appropriate, several factors such as the brine volume; chemical characteristics, capital and operating costs, and the capacity of storage and transport should be take into consideration [11]. Brine treatment options are grouped into four different categories according to the final use [16,17]: (1) zero liquid discharge (ZLD) [18]: (2) technologies for industrial salt recovery; (3) adaptation of brine for industrial applications; and (4) metal recovery. In all of those methods, the environmental effects of the ecosystem, in the case of seawater RO plants, and groundwater aquifers deterioration, in case of inland RO plants, should take into consideration. Table 1 shows the different techniques of brine disposal treatment. From the table, the higher cost is shown in the case of evaporation ponds, while the lower one was with the sea surface discharge that environmentally undesirable. With regard to inland RO desalination plants, the costs associated with brine processing may be considerably higher than that of coastal plants, depending on the salinity of the brine concentrate. As illustrated in Table 1, several disposal solutions have been used, in particular for inland desalination plants, some of which include discharge into solar evaporation ponds, disposal into wastewater systems, land applications, injection into a deep saline groundwater aquifer, land disposal, and disposal into the sea by long pipeline systems [4,11,19,20].

Nanofiltration is a process between RO and ultrafiltration which is powered by pressure. The key benefits are lower working pressure and greater divalent cations rejection than monovalent ones. NF is a method used to extract divalent salts that cause water hardness and scaling onto the surface of the RO membranes (calcium, magnesium, and barium salts, etc.) [28]. In other words, NF can work as a conventional water softener but without the requirement for regeneration. NF has the superior rejection of polyvalent cations without stoichiometric chemical dosing for the deposition of components of hardness and, consequently, the lack of sludge formation, as opposed to softening. Moreover, NF results in partial desalination of the brine stream, while softening results in a rise in the concentration of some components, such as sodium and carbonate ions, and does not change other components that are not involved in the softening reactions, such as SO_4_^2−^ and Cl^−^ ions, increasing overall total dissolved salts (TDS). NF membrane process plays an important role in water treatment; because it can operate at low operating pressure with lower permeate flux and high divalent and multivalent ion salts retention [29,30]. Additionally, it is the most technical and economical solution for removing sulfates from the brine. NF can be selected with a high affinity to the rejection of sodium sulfate, allowing a more effective recirculation of the brine [31,32]. 

NF has the advantages of ease of operation, durability, comparatively low energy consumption, and highly efficient elimination of contaminants. Reducing the use of additives in pre-treatment procedures, as well as reducing the expense of energy usage and water processing, has resulted in more environmentally sustainable processes. Besides, where the evaporation rate is low or the drainage rate is high, evaporation ponds require vast tracts of land, the need for impermeable liners of clay or plastic membranes such as PVC, and the ability to contaminate underlying potable water aquifers by infiltration from improperly built evaporation ponds. NF membranes have recently been used by these techniques, as they have fewer environmental risks [14,33,34,35]. 

Brine discharge produced from inland desalination plants represents a critical issue in concern of groundwater aquifers deterioration. The groundwater chemical type is one of the main factors affecting desalination and brine treatment process. Therefore, detailed study of water characteristics is very important to determine the treatment process. In this work, RO brine treatment (related to water composition) was carried out using a commercial NF, as a pretreatment process, in order to decrease the scaling potential on TFC-RO membranes via removal of divalent ions. The salt scale formation on the membrane surface is supposed to be low because NF could work at a high recovery level; its use in the pre-treatment of RO brine could substantially render the NF permeated water ideal for a further RO step, which minimizes energy usage and hence the overall total costs. RO brine discharge from two desalination plants (Brackish groundwater and Seawater) was used as feed water in the NF process, where the efficiency and selectivity of the NF membrane were carried out. To predict the performance of NF membranes for pre-treatment and minimizing the inorganic fouling on RO membrane surfaces, hypothetical (predominant) soluble salt estimation, prediction, and knowledge of the salts present in feed water samples during the desalination process was very helpful during process operation. To achieve the goal of this work, a commercial a NF membrane element with high divalent salt rejection was used.

## 2. Materials and Methods

### 2.1. Materials

In this work, a commercial spiral-wound nanofiltration membrane element (NF-2012-250, Polyamide Thin-Film Composite membranes, provided by Soul-water Co., Cairo, Egypt) was used. The membrane active area (0.56 m^2^), pore size ranges from 1 to 10 nm permeate flow rate (250 gallons per day), 30–50% NaCl rejection (500 mg/L), 95% MgSO_4_ rejection (2000 mg/L) at 0.4 MPa [36]. Salts, such as magnesium sulfate, magnesium chloride, sodium sulfate, and sodium chloride, have been used to assess membrane efficiency and purchased from a local company (Elgomhoryia Co.), Cairo, Egypt. All salts are analytical grade (purity, about 99%) and were used without purification.

### 2.2. Chemical Characterization of RO Feeds and Brines

In this study, four water samples obtained from brackish and seawater desalination plants [brackish groundwater (BW) and its brine (BW-brine) concentrates (Shammas desalination plant with a capacity of 100 m^3^/day), seawater (SW), and its brine (SW-brine)] concentrate, Elremeala, 24,000 m^3^/day, were collected and extensively analyzed in terms of chemical constituents, Table 2. Laboratory analyses of the samples included the determination of the electrical conductivity (EC), pH, and concentration of the major ions were carried out. EC and pH were measured by a conductivity meter (Model LF 538, WTW, Arlington, VA, USA) and pH meter (3510, Jenway, Staffordshire, UK) respectively. Major ions (Ca^2+^, Mg^2+^, CO_3_^2−^, HCO_3_^−^, SO_4_^2−^ and Cl^−^) were calculated using the standard method of titration, while Na^+^ and K^+^ were measured by a flame photometer [37,38,39,40]. The Total dissolved salts (TDS) measured in mg/L were calculated from Equation (1);

(1) $$\mathrm{TDS}=\;[{\mathrm{Ca}}^{2+}]+\;[{\mathrm{Mg}}^{2+}]+[{\mathrm{Na}}^{+}]+[K^{+}]+[{\mathrm{CO}}_{3}^{2-}]+[{\mathrm{HCO}}_{3}^{-}]+[{\mathrm{SO}}_{4}^{2-}]+[{\mathrm{Cl}}^{-}]$$

The Ionic balance (IB), the difference between the concentrations of total cations (TC) and total anions (TA) that must be within ±5, was calculated according to Equation (2);

(2) $$\mathrm{IB}=\left[\frac{\mathrm{TC}-\mathrm{TA}}{\mathrm{TC}+\mathrm{TA}}\right]\times \;100$$

The total hardness (TH), in mg/L, was calculated from Equation (3) [41];

(3) $$\mathrm{TH}=2.5\left({\mathrm{Ca}}^{2+}\right)+4.1\left({\mathrm{Mg}}^{2+}\right)$$

The saturation indices (SI) of the major mineral phases in the water samples under investigation were determined using the NETPATH-WIN software package as illustrated in Equation (4) [42];

(4) $$\mathrm{SI}=\log\left(\frac{K_{IAP}}{K_{So}}\right)$$

*K_IAP_* and *K_So_* are the ionic activity and solubility products, respectively, of mineral dissolution at a given temperature. If the SI is equal to zero, the water is in equilibrium or saturated with the mineral phase, the SI value below zero (negative value) indicates under-saturation and the mineral phase tends to dissolve, while the SI value above zero (positive value) indicates super-saturation and the mineral phase tends to precipitate.

The Langelier Saturation Index (LSI) is one of the most widely used calcium carbonate scaling tendency indices. It is obtained from the Reverse Osmosis System Analysis (ROSA) software program. When the LSI value is negative, calcium carbonate will not precipitate; when LSI is equal to zero, calcium carbonate will saturate the solution; if LSI is positive, calcium carbonate will form a scale [43,44].

Estimation of the Hypothetical electrolytes concentration; Hypothetically, strong acid ions (Cl^−^ and SO_4_^2−^) may form a chemical combination with strong alkali (Na^+^ and K^+^) in any water supply, whereas the rest of the acid radicals combine with the alkaline earth (Ca^2+^ and Mg^2+^). If the alkali and alkaline earth cations are excessive, they can react with the weak acid (CO_3_^2−^ and HCO_3_^−^) anions [45,46]. The relations between cations and anions in the waters investigated are illustrated. Based on calculations five hypothetical soluble salts (NaCl, MgCl_2_, CaCl_2_, CaSO_4_, and Ca(HCO_3_)_2_) are found.

**Table 2:** Chemical characterization of the RO feeds and brines from two desalination plants: Shammas (brackish water) and El-Remela (Seawater), Matrouh governorate, Egypt.

Parameter	Brackish Water (Groundwater Well)		Seawater (Mediterranean Sea)	
	Feed	Brine	Feed	Brine
pH	8.2	8	8	8.1
TDS (mg/L)	5881.4	15,115.9	39,831.1	57,097.2
Ca^2+^ (mg/L)	184	320	450	560
Mg^2+^ (mg/L)	194.4	583.2	860	1919.7
Na^+^ (mg/L)	1700	4600	13,000	18,400
K^+^ (mg/L)	37	110	430	560
CO_3_^2−^ (mg/L)	12	18	24	30
HCO_3_^−^ (mg/L)	183	311.1	134.2	176.9
SO_4_^2−^ (mg/L)	360	1837	2200	3500
Cl^−^ (mg/L)	3302.5	7492.2	22,800	32039
Ca^2+^ hardness (mg/L)	460	800	1125	1400
Mg^2+^ hardness (mg/L)	800	2400	3539.1	7900
TH (mg/L)	1257.04	3191.12	4651	9270.77
NaCl (mg/L)	4423.4	11,493.11	39,856.21	46,488.75
MgCl_2_ (mg/L)	837.81	1021.97	475.75	6140.6
MgSO_4_ (mg/L)	106.87	1695.98	2523.51	2872.41
CaSO_4_ (mg/L)	350.516	567.34	671.41	1368.22
Ca(HCO_3_)_2_ (mg/L)	192.01	337.53	174.02	227.08
SO_4_^2−^/HCO_3_^−^	1.25	3.75	10.41	12.57
Calcite	0.851	0.925	0.602	0.837
Aragonite	0.707	0.781	0.458	0.693
Dolomite	2.087	2.479	1.886	2.627
Gypsum	−1.263	−0.66	−0.679	−0.517
anhydrite	−1.48	−0.873	−0.879	−0.708
LSI	0.46	0.75	0.036	0.48

### 2.3. Experimental Set-Up and Membrane Performance Evaluation 

**Figure 1:**
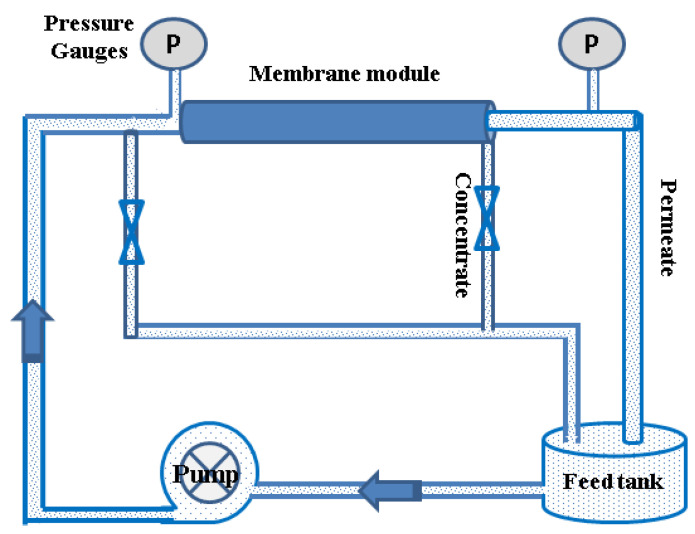
Schematic representation of the NF system.

All membrane experiments were carried out in a laboratory test cell using a homemade test unit module, Figure 1. NF-2012-250, USA membrane can reject monovalent ions at relatively low values and divalent ions at reasonable values and reduced seawater salinity to 33 g/L at very high permeate flux. The permeate and brine flow rates were 15 and 5 LPM, respectively, with a recovery ratio of about 75%. Both permeate and the brine recycled to the feed tank. 

The selectivity of the membrane element was depicted using an aqueous solution of separate salts of NaCl, MgCl_2_, MgSO_4_, CaCl_2_, and CaSO_4_. During the experiment, solutions of permeate and reject were returned to the feed tank to ensure a constant concentration of the feed solution. Both salt rejection (*R_s_* %) and water flux (*J_w_*) were determined using Equations (5) and (6) as follow;

(5) $$R_{s}\;\%=\left(1-\frac{C_{p}}{C_{f}}\right)\times 100$$

(6) $$J_{w}\;=\;\frac{V}{A\cdot t}$$

*C_p_* and *C_f_*, the concentration (mg/L) of permeate and feed solutions, respectively, were determined using a conductivity meter (Model LF 538, WTW, USA). *J_w_* is the water flux in L/m^2^·h, *V* is the permeate volume [L]; A is the membrane area in [m^2^], and *t* is the permeation time [h].

## 3. Results

### 3.1. Chemical Analysis of the Water Samples

During the membrane-based desalination process, the characteristics of the feed water including TDS, type and concentration of ions, type of salts, total hardness, and degree of saturation indices have an effect on the membrane performance and recovery percentage as well as the salt concentration of the brine discharge. As illustrated in Table 2, the concentration of salts in brine water mainly depends on the desalination plant recovery rate. BWRO-brine’s high salinity of 15,115.9 mg/L out of 5881.4 mg/L is attributed to the higher rate of recovery of the desalination plant (~80%) compared to that of seawater (~30%). 

Scaling onto the surface of RO membranes has become a critical issue in operating processes as it causes a decline in water flux, damage to the membrane, and high energy consumption [47]. In any RO process, divalent salts are rejected more than monovalent, i.e., the concentrations of calcium, magnesium, and sulfate ions in the brine concentrate are very high and lead to increased TH values, which were 3191.12 and 9270.77 mg/L of both BWRO and SWRO, respectively. Moreover, due to the high concentrations of calcium and magnesium hardness (800 and 2400 mg/L of BWRO and 1400 and 7900 mg/L of SWRO reject, respectively), and the degree of saturation indices, scale deposition on the membrane surface should be established. 

The estimation of soluble salts composition in groundwater feed or brine discharge to any desalination plant contributes to a greater extent to the life-time and specifications needed for RO plant establishment. The combination of the major cations and anions indicates the formation of five primary hypothetical electrolytes in all feed and brine samples, Table 2. 

Table 2 indicates both salt concentration and type alerts after desalination by RO. A substantial increase in the concentration of MgSO_4_ was observed in BWRO Rejection (1695.98 out of 106.87 mg/L) and SWRO Rejection (2872.41 out of 2523.51 mg/L). Besides, the concentration of sodium chloride rose from 4423.4 to 11,493.11 mg/L in BWRO brine concentrate and increased from 39,856.21 to 44,688.75 mg/L in SWRO. Increased values of CaSO_4_ and MgSO_4_ in concentrations of brackish water desalination brine and CaSO_4_ in desalination with seawater require the use of a pre-treatment step is a required step to avoid scaling on the surface of the membrane. 

### 3.2. Evaluating the Performance of the NF Process

First, to characterize the efficiency of the NF element, different salt solutions were used as a feed solution at different applied pressures. Table 3 shows the influence of trans-membrane inlet pressures on the water flux and the salt rejection of various synthetic salt solutions. Table 3 shows a pressure dependence of all salts water fluxes that generally increased with increases in inlet pressure with an insignificant shift in salt rejection, except that of sodium chloride. The NF membrane element achieved higher water flux with a solution of sodium chloride (0.79 m^3^/day at 7 bar) while the lower flux accompanied desalination of MgSO_4_ solution (0.3 m^3^/day), this was expected as the larger size of divalent ions could block the membrane pores. On the other hand, salt rejection follows the order of CaSO_4_ ≥ Na_2_SO_4_ > MgSO_4_ > MgCl_2_ > NaCl, i.e., salts with divalent ions were rejected more than monovalent salt. The membrane demonstrated strong Magnesium Salt Rejection, mild calcium salt Rejection, and low NaCl Salt Rejection. This rejection activity can be demonstrated by the fact that the membrane seems to be positively charged in the presence of MgSO_4_, MgCl_2_, CaSO_4_, and Ca(HCO_3_)_2_ salts and negatively charged in the presence of NaCl. These findings suggest that each ion may have an individual contribution to the membrane load by adsorption, this phenomenon is due to ionic adsorption. In the case of MgSO_4_, MgCl_2_, CaSO_4,_ and Ca(HCO_3_)_2_ salts, there is heavy adsorption of Mg^2+^ and Ca^2+^ ions resulting in reverence of the membrane load so that the membrane is positively charged. This may justify the fact that these salts are better rejected than NaCl. This rejection sequence is very well suited with earlier works on NF membranes that achieved very high rejection of divalent anions with a minimal rejection of monovalent ions and NF targets [48,49,50,51].

**Figure 2:**
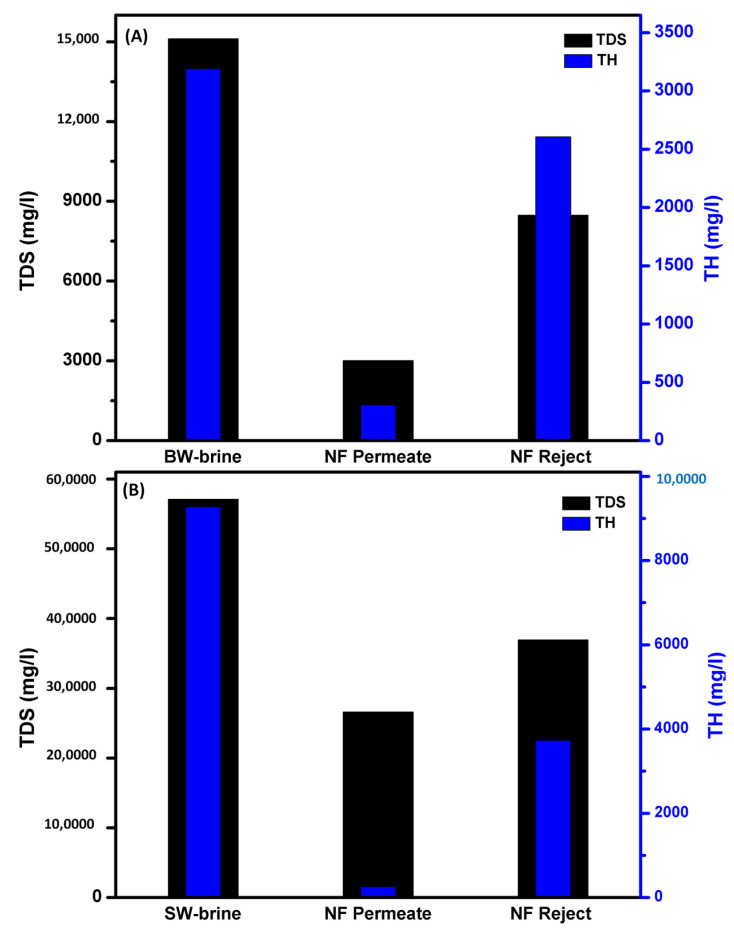
Total dissolved salts and total hardness removal percentages using the membrane of (**A**) Brackish water, and (**B**) Sea water. operating conditions: pressure 0.7 MPa, operation time 8 h, operation temperature, 25 °C & flow rate, 5 L/min.

Using NF as a pre-treatment process, the TDS of BWRO and SWRO brine decreased from 15,115.9 and 57,097.2 mg/L to 3009.8 and 26,838.1 mg/L, with a rejection rate of 81% and 53%, respectively, as shown Figure 2. The low salt rejection of the NF element for SWRO reject is due to the application of a limited applied pressure (7 bar) due to the lab system used, in addition to the effect of ion repulsion at higher feed concentrations [52]. TH decreased from 3191.1 and 9270.77 mg/L to 304.9 and 259.5 mg/L, with a removal percent of 90.4 and 97.2% for BWRO and SWRO, respectively. This high rejection of TH shows the higher affinity of the NF membrane for removing divalent ions.

**Figure 3:**
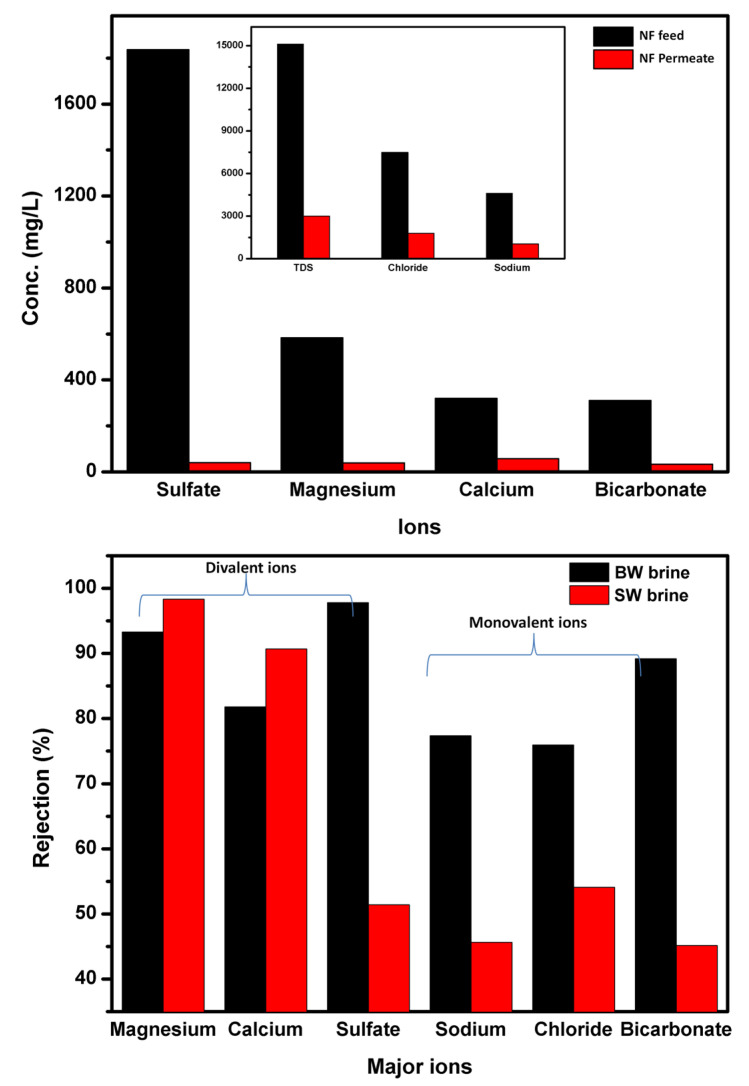
Removal of divalent and monovalent ions from BWRO and SWRO brines using the membrane operating conditions: pressure 0.7 MPa, operation time 8 h, operation temperature, 25 °C & flow rate, 5 L/min.

NF is known to have a very high degree of rejection of divalent ions with a limited rejection of monovalent ions depending on its pore structure. Figure 3 shows NF membrane behavior against the treatment of BWRO and SWRO brine concentrates as well as the parameters of the major ion. From the Table 4, the NF element showed higher rejection of divalent ions due to the larger ion and hydrated ion radii than that of monovalent ions. Low rejection of monovalent ions is due to their higher diffusivity, ions are more rejected if they have a lower diffusivity, in addition to hydration energy that plays an important role in rejection, the more hydrated the divalent ions, the more difficult their transfer through the membrane [48], therefore, the rejection of ions from BWRO and SWRO feed concentrates follows the order of R_SO4_ > R _HCO3_ > R_Cl_ and R_Mg_ > R_Ca_ > R_Na_. SO_4_^2−^ and HCO_3_^−^ ions, which are more strongly hydrated than Cl^−^ ions, become difficult to penetrate through the membrane. The observed low rejection of the anions of SWRO is due to that high feed concentration leading to the formation of a screen phenomenon that inhibits the Donnan effect of electrical repulsion between the negatively charged membrane surface and the anions [53]. The higher rejection of the ions, except calcium and magnesium, of BWRO brine compared to SWRO brine is due to that at a higher feed concentration, the rejection ratio of any salt is lower due to the lower Donnan exclusion by the membrane [54].

SO_4_^2−^/HCO_3_^−^ molar ratio is used to estimate the propensity for carbonate fouling onto the surface of membranes [55]. The high ratio values of BWRO and SWRO brines, 3.75 and 12.57, respectively, indicate a moderate and very high carbonate scaling potential. These values decreased to 0.75 and 11.13, respectively, after NF treatment as the process reduce scale formation onto the next stage of RO. It is important to mention that scale forming depends on the process recovery, as NF should work at high recovery (about 80%) therefore the passage of the salt would be very high with a low tendency of scale formation.

**Figure 4:**
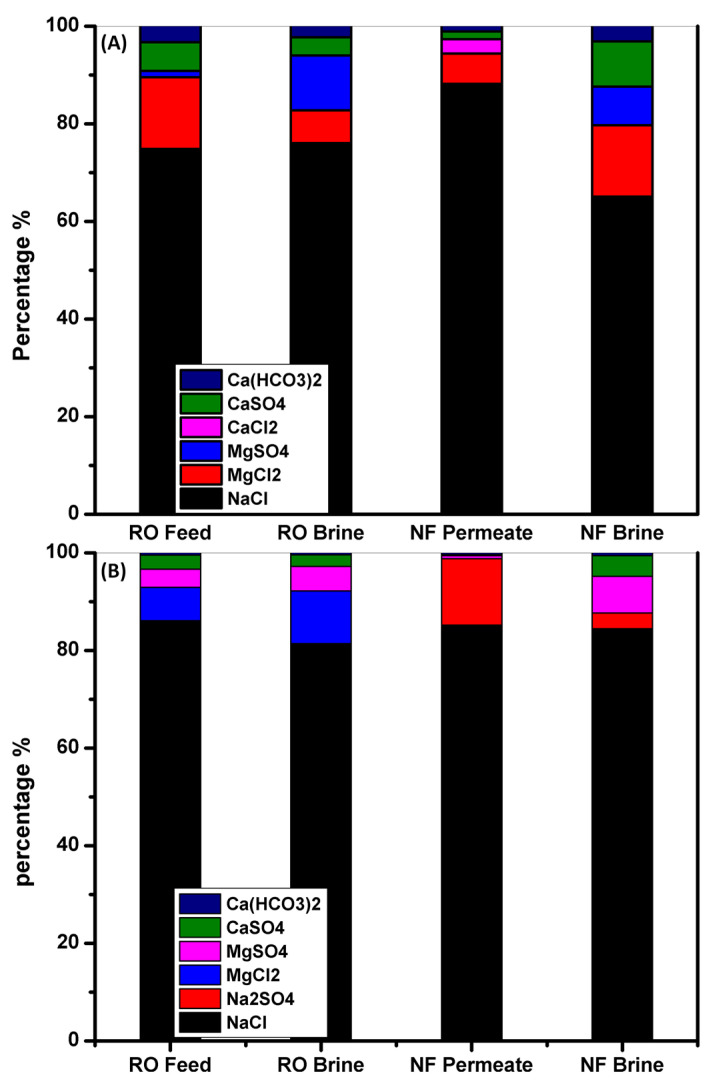
Bar graph representing the hypothetical ions combination (electrolyte) of the feed, RO-brine, NF permeate, and NF brine of (**A**) BW and (**B**) SW samples. Operating conditions: pressure 0.7 MPa, operation time 8 h, operation temperature, 25 °C & flow rate, 5 L/min.

The hypothetical electrolytes in BWRO and SWRO brine concentrates, NF permeates, and NF reject was determined based on the percentage molar ratios of the ions, Table 5 and Figure 4, to elucidate the action of NF for desalination of mixed salt solution. The findings show that after the NF phase, the concentrations and, in certain cases, the form of salt have changed. There was a rise in the concentration of sodium chloride in all BWRO and SWRO brine concentrates, with a decline in all divalent salt concentrations (Na_2_SO_4_, MgSO_4_, and CaSO_4_) and a new presence of sodium sulfate with the absence of magnesium chloride in NF permeate. During the desalination process, the presence of CaSO_4_ in the hypothetical electrolyte is due to the rejection of more sulfate ions than calcium ions, because of the excess calcium interacted with chloride ions to maintain balance. Out of Figure 3, the low concentration of divalent salts makes this water ideal as a feed to the next stage of a RO phase with less scaling formation.

**Figure 5:**
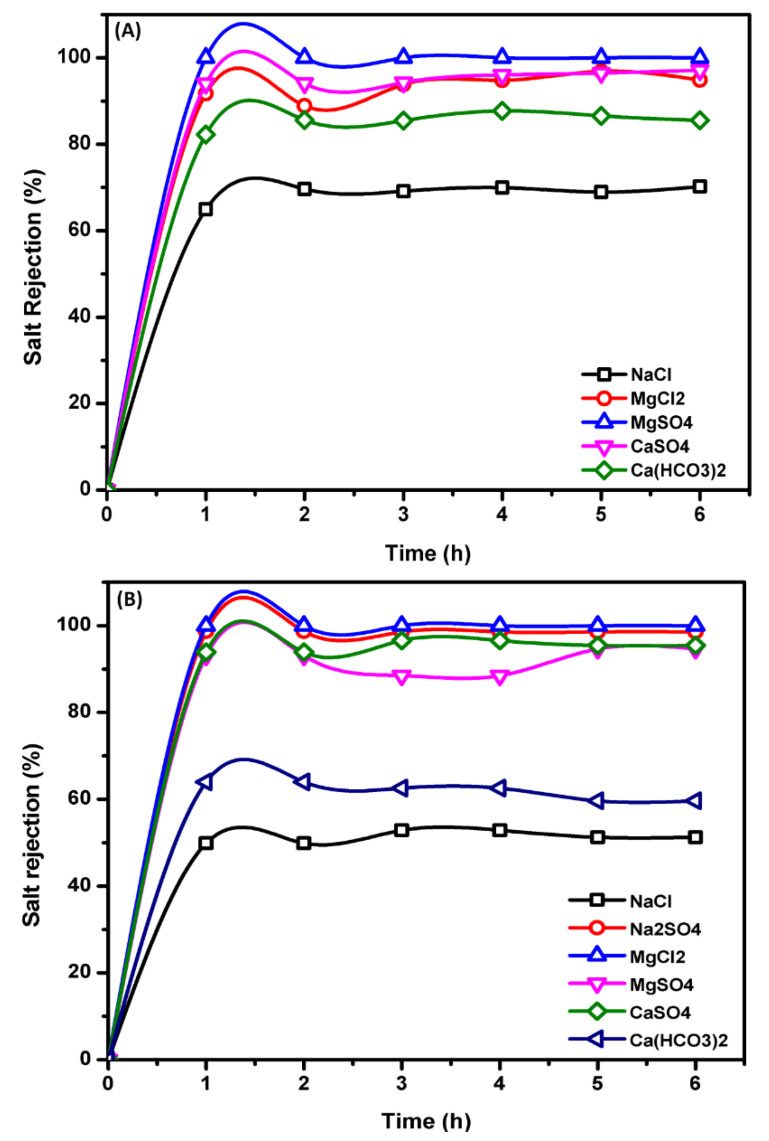
Hypothetical electrolytes rejection using NF membrane element of BWRO (**A**) and SWRO (**B**) brine concentrate operating conditions: pressure 0.7 MPa, operation time 8 h, operation temperature, 25 °C & flow rate, 5 L/min.

Figure 5 shows that the salt rejection of the NF membrane for the predicted hypothetical electrolytes was as follows; MgSO_4_ > CaSO_4_ > MgCl_2_ > Ca (HCO_3_)_2_ > NaCl. The higher rejection of sulfate salts more than chloride salts is due to the increased Donnan exclusion of divalent ions compared to that of the monovalent Cl^−^ ion. The higher rejection of MgCl_2_ compared to NaCl is because of two factors; the first is the salt diffusivity (diffusion coefficient) Table 4, where NaCl has a diffusivity of 1.48 × 10^−9^ m^2^·s^−1^ greater than that of MgCl_2_, 1.04 × 10^−9^ m^2^·s^−1^, respectively [56,57]. Therefore, NaCl salt ions are more permeable through the membranes than MgCl_2_ salt ions. The second factor is based on the charge of the membrane, where the interaction of magnesium and calcium ions onto the membrane surfaces converts the membrane surface charge from negative to positive so that the surface of the membrane is positively charged. This may clarify the fact that in the case of MgCl_2_, this salt is better rejected than NaCl, where Mg^2+^ is the co-ions (an ion with the same load sign as the membrane load) and have a larger valence than the chloride, which is the co-ion in the case of NaCl. According to the principle of Donnan exclusion, a strong co-ion valence induces greater salt rejection. In brackish and saline groundwater samples, the greater rejection of CaSO_4_ than Ca (HCO_3_)_2_ is due to the negatively charged membrane, so the higher valence co-ions SO_4_^2−^ are highly rejected compared to HCO_3_^−^ [58]. In the case of SWRO brine, owing to the low concentration of calcium bicarbonate in the feed solution (281.63 mg/L), Ca (HCO_3_)_2_ is more rejected than CaSO_4_.

Saturation indices; while precipitation and fouling of compounds precipitate on RO membrane surfaces has been studied significantly over the past years, less focus has been paid to their formation in the reject brine during the RO process. A rise in saturation index above 1 could contribute to the precipitation of dissolved minerals and the formation of salts layer in the RO brine, which could cause fouling problems on membranes. This will seriously impair water transport efficiency and reduce the value of the overall membrane [59]. The determined LSI of the BWRO and SWRO brines are 0.75 and 0.48, respectively, meaning that this water is supersaturated concerning calcium carbonate and scale formation which occur. After NF, LSI values were found to be less than zero (−0.34 and −0.52), i.e., water is unsaturated with calcium carbonate. 

**Figure 6:**
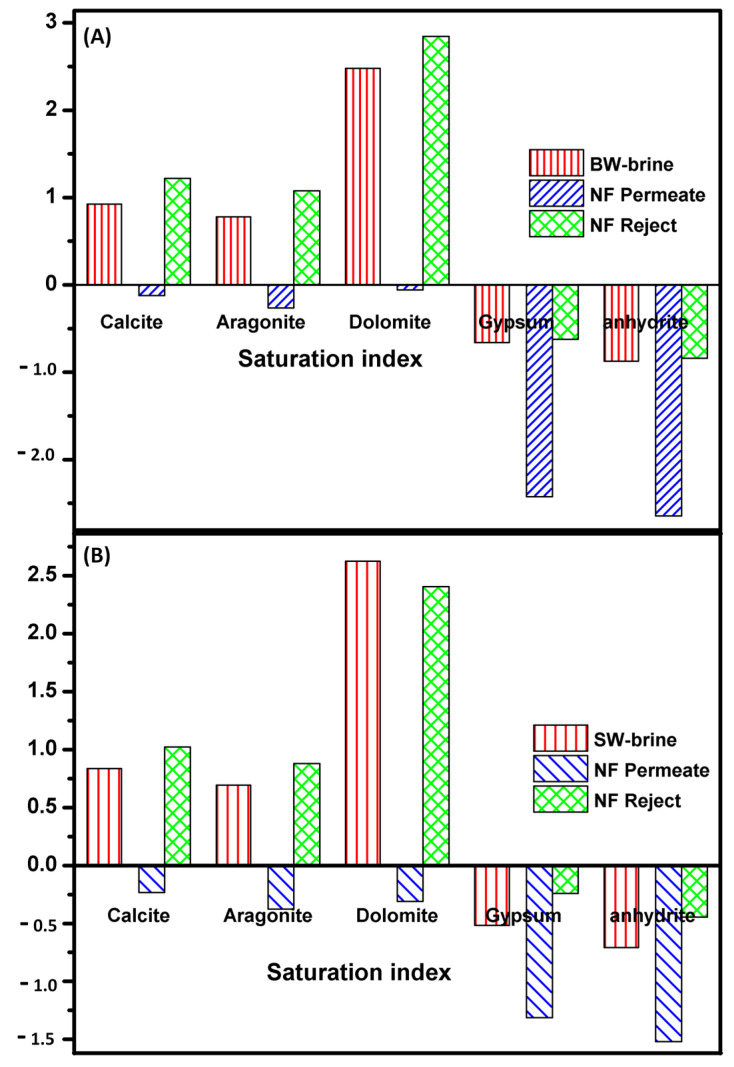
Saturation indices of RO-brine, NF permeate and NF brine of (**A**) BW and (**B**) SW samples operating conditions: pressure 0.7 MPa, operation time 8 h, operation temperature, 25 °C & flow rate, 5 L/min.

Figure 6 reflects that BWRO and SWRO brine have positive values of calcite, aragonite, and dolomite indices indicating a slight super-saturation. This means that in case of treatment of the brine concentrated produced from desalination of the collected samples it is important to make a pretreatment step to prevent scaling. 

Uses of NF brine discharge; As NF is implemented, the procedure should be physically and economically viable and the most effective and economical process design choices and possibilities should be defined. The cost of the NF process depends primarily on the configuration of the assembly, membrane flow, device operating conditions and plant capacity. As NF removes the divalent ions more effectively than monovalent ions, therefore NF brine with high concentrations of calcium and magnesium concentrations can be used for different industries as discussed in a previous work [60]. For example, in drinking water treatment plants, magnesium ions can be applied to post-treated desalinated water, while in wastewater treatment applications, magnesium phosphate species can be extracted by precipitation of magnesium phosphates, magnesium phosphates (hydroxyapatite), or magnesium/calcium phosphates, which are theoretically reusable as new phosphorate materials. There have also been studies of the use of Mg^2+^-rich brines for the processing of magnesium sulfate by incorporation of a concentration phase through membrane distillation and crystallization formation onto membrane surfaces. Technologies, including surface water discharge, deep well injection, and evaporation ponds are unsustainable and their usage is constrained by the high cost of capital and minimal implementations [10]. NF observed superior rejection of polyvalent cations from the reject source, along with the absence of chemical dosing stoichiometric to deposition of hardness components and, consequently, absence of sludge formation, which is a regular disposal issue such as that of lime softening. 

**Table 4:** Some parameters and rejection percentages of the ions in water and their rejection using NF membrane [48,61,62].

Ion	Diffusivity (10^−9^ m^2^·s^−1^)	Ionic Radius (nm)	Hydrated Ionic Radius (nm)	Hydration Energy (kJ·mol^−1^)	Rejection BW-Brine (%)	Rejection SW-Brine (%)
Mg^2+^	0.706	0.074	0.429	1921	93.3	98.35
Ca^2+^	0.92	0.099	0.349	1584	81.8	90.71
Na^+^	1.333	0.095	0.365	407	77.39	45.65
SO4^2−^	1.065	0.230	0.380	1138	97.82	51.42
HCO_3_^−^	1.85	--	--	--	89.21	45.16
Cl^−^	2.032	0.181	0.347	376	75.97	54.11

**Table 5:** Hypothetical ions (in mg/L) combination and their removal using NF membrane.

Hypothetical Electrolytes	BW			SW		
	BWRO Brine	NF Permeate *	NF Brine	SWRO Brine	NF Permeate *	NF Brine
TDS	15,115.9	3009.775	11,652.7	58,547.55	26,611.74	36,934
NaCl (mg/L)	11,493.11	2326.6	7863.65	46,488.7	21,522.43	29,528.8
Na_2_SO_4_ (mg/L)	0	6.20	53.92	0	4875.07	4286.99
MgCl_2_ (mg/L)	1021.97	227.90	0	6140.67	0	0
MgSO_4_ (mg/L)	1695.98	0	1879.16	2872.41	74.69	1514.72
CaCl_2_ (mg/L)	0	88.62	0	0	0	0
CaSO_4_ (mg/L)	567.344	48.02	1480.73	1368.22	0	1316.75
Ca(HCO_3_)_2_ (mg/L)	337.53	31.74	375.22	227.08	133.26	286.68

* Note; the summation of the salts of NF permeate and reject not equal that of RO reject feed water, because of deposition of some salts onto membranes surface.

## 4. Conclusions

A nanofiltration (NF) procedure was used in this study for the removal of divalent ions from the brine discharge. The obtained results show that the salt rejection of NF membrane follows the order of CaSO_4_ (97.4%), Na_2_SO_4_ (97.3%), MgSO_4_ (95.2%), MgCl_2_ (93.4%), and NaCl (79%), respectively. The results show that 79–89% of total dissolved salts (TDS) and 96–98% of total hardness (TH) retained by this NF process. The rejection of divalent ions by NF was in the order of sulfate (>95%), magnesium (>60%), and calcium (>30%) in every rejection experiment based on water recovery rate (40, 50, 60, 70, and 80%). 

## Figures and Tables

**Table 1:** Different technologies of RO-brine treatment.

Brine Treatment Technique	Cost (US$/m^3^)	Advantages	Disadvantages	Reference
Surface Water Discharge	0.05–0.3	Available and cost-effective for all desalination plants	Damage of ecosystem, discharge of chemicals of membranes cleaning	[21]
Fixed bed column softener	--	highly efficient with relatively low cost and energy requirements	Degradation and oxidation in some cases, discharge of excess NaCl to the aquatic environment	[22]
Solar concentrators (solar ponds)	--	Low cost, a valuable by-product	Requires a lot of maintenance, requires large land areas, needs adequate sunlight	[23]
Evaporation Ponds	3–10	Salt production, low maintenance and little operator attention	High footprint and costs,	[24]
Land Applications	0.74–1.95	Easy operation and implementation	limited to small plants	[2]
Deep Well Injection	0.54–2.65	Low energy consumption, moderate cost	Groundwater contamination	[2]
Sewer Discharge	0.32–0.66	Low cost and energy consumption, easy implementation	Limited to small size flows	[25]
Membrane-based technology (HP-RO)	0.75–0.79	High production of desalted water	High cost because of frequently membranes replacement	[26]
Forward osmosis	0.63	Efficient with high salt content	Low production of water	[27]
Thermal-based technology	0.09–1.2	Efficient with high salt content	High energy consumption	[2]
This work (NF-based hypothetical electrolytes prediction)	--	Ease of operation, durability, low energy consumption, and highly efficient elimination of contaminants	Membranes scaling	--

**Table 3:** The water flux (*J_w_*, L/day) and salt rejection (%) of the NF membrane element as a function of the applied pressure, salt concentration is 2000 mg/L.

Salt	MgSO_4_		Na_2_SO_4_		CaSO_4_		MgCl_2_		NaCl	
Pressure (bar)	*J_w_*	*R_s_*	*J_w_*	*R_s_*	*J_w_*	*R_s_*	*J_w_*	*R_s_*	*J_w_*	*R_s_*
4	46	92	72	96.2	246.8	96	216	90.5	320	68
5	100.8	93.7	370.2	97.4	481.3	97	376.4	92.5	530	73
7	308.4	95.2	709.7	97.3	752.91	97.4	709.7	93.4	790	79

## Data Availability

Data sharing not applicable.

## References

[1] Du J.R., Zhang X., Feng X., Wu Y., Cheng F., Ali M.E. (2020). Desalination of high salinity brackish water by an NF-RO hybrid system. Desalination.

[2] Panagopoulos A., Haralambous K.-J., Loizidou M. (2019). Desalination brine disposal methods and treatment technologies—A review. Sci. Total Environ..

[3] Pangarkar B.L., Sane M.G., Guddad M. (2011). Reverse Osmosis and Membrane Distillation for Desalination of Groundwater: A Review. ISRN Mater. Sci..

[4] Mohamed A., Maraqa M., Al Handhaly J. (2005). Impact of land disposal of reject brine from desalination plants on soil and groundwater. Desalination.

[5] Mezher T., Fath H., Abbas Z., Khaled A. (2011). Techno-economic assessment and environmental impacts of desalination technologies. Desalination.

[6] Ahmed M., Shayya W.H., Hoey D., Al-Handaly J. (2001). Brine disposal from reverse osmosis desalination plants in Oman and the United Arab Emirates. Desalination.

[7] Younos T. (2009). Environmental Issues of Desalination. J. Contemp. Water Res. Educ..

[8] Qiu T., Davies P.A. (2012). Comparison of Configurations for High-Recovery Inland Desalination Systems. Water.

[9] Istirokhatun T., Dewi M., Ilma H., Susanto H. (2018). Separation of antiscalants from reverse osmosis concentrates using nanofiltration. Desalination.

[10] Arnal J., Sancho M., Iborra I., Gozálvez J., Santafé A., Lora J. (2005). Concentration of brines from RO desalination plants by natural evaporation. Desalination.

[11] Pramanik B.K., Shu L., Jegatheesan V. (2017). A review of the management and treatment of brine solutions. Environ. Sci. Water Res. Technol..

[12] Tao G., Viswanath B., Kekre K., Lee L.Y., Ng H.Y., Ong S.L., Seah H. (2011). RO brine treatment and recovery by biological activated carbon and capacitive deionization process. Water Sci. Technol..

[13] Ji X., Curcio E., Al Obaidani S., Di Profio G., Fontananova E., Drioli E. (2010). Membrane distillation-crystallization of seawater reverse osmosis brines. Sep. Purif. Technol..

[14] McGinnis R.L., Hancock N.T., Nowosielski-Slepowron M.S., McGurgan G.D. (2013). Pilot demonstration of the NH3/CO2 forward osmosis desalination process on high salinity brines. Desalination.

[15] Liu J., Yuan J., Ji Z., Wang B., Hao Y., Guo X. (2016). Concentrating brine from seawater desalination process by nanofiltration–electrodialysis integrated membrane technology. Desalination.

[16] Morillo J., Usero J., Rosado D., El Bakouri H., Riaza A., Bernaola F.-J. (2014). Comparative study of brine management technologies for desalination plants. Desalination.

[17] Afrasiabi N., Shahbazali E. (2011). RO brine treatment and disposal methods. Desalin. Water Treat..

[18] Semblante G.U., Lee J.Z., Lee L.Y., Ong S.L., Ng H.Y. (2018). Brine pre-treatment technologies for zero liquid discharge systems. Desalination.

[19] Ahmed M., Shayya W.H., Hoey D., Mahendran A., Morris R., Al-Handaly J. (2000). Use of evaporation ponds for brine disposal in desalination plants. Desalination.

[20] Ünlü K., Kemblowski M., Parker J., Stevens D., Chong P., Kamil I. (1992). A screening model for effects of land-disposed wastes on groundwater quality. J. Contam. Hydrol..

[21] Arafat H. (2017). Desalination Sustainability: A Technical, Socioeconomic and Environmental Approach.

[22] Al-Ghamdi A.A. (2017). Recycling of Reverse Osmosis (RO) Reject Streams in Brackish Water Desalination Plants using Fixed Bed Column Softener. Energy Proced..

[23] Shalaby M., Abdalla H.S., Shaban A., Mohamed H.A., Mohamed W.A. (2020). Brine Treatment by Solar Energy: Case Study in Tor-Sinai—Egypt. Egypt. J. Chem..

[24] Dama-Fakir P., Toerien A. Evaporation rates on brine produced during membrane treatment of mine water. Proceedings of the International Mine Water Conference.

[25] Chang J.-S. (2015). Understanding the role of ecological indicator use in assessing the effects of desalination plants. Desalination.

[26] Schantz A.B., Xiong B., Dees E., Moore D.R., Yang X., Kumar M. (2018). Emerging investigators series: Prospects and challenges for high-pressure reverse osmosis in minimizing concentrated waste streams. Environ. Sci. Water Res. Technol..

[27] Linares R.V., Li Z., Yangali-Quintanilla V., Ghaffour N., Amy G., Leiknes T., Vrouwenvelder J. (2016). Life cycle cost of a hybrid forward osmosis-low pressure reverse osmosis system for seawater desalination and wastewater recovery. Water Res..

[28] Bowen W., Mukhtar H. (1996). Characterisation and prediction of separation performance of nanofiltration membranes. J. Membr. Sci..

[29] Hafiane A., Lemordant D., Dhahbi M. (2000). Removal of hexavalent chromium by nanofiltration. Desalination.

[30] Van Der Bruggen B., Koninckx A., Vandecasteele C. (2004). Separation of monovalent and divalent ions from aqueous solution by electrodialysis and nanofiltration. Water Res..

[31] Meihong L., Sanchuan Y., Yong Z., Congjie G. (2008). Study on the thin-film composite nanofiltration membrane for the removal of sulfate from concentrated salt aqueous: Preparation and performance. J. Membr. Sci..

[32] Krieg H., Modise S., Keizer K., Neomagus H. (2005). Salt rejection in nanofiltration for single and binary salt mixtures in view of sulphate removal. Desalination.

[33] Chidambaram T., Oren Y., Noel M. (2015). Fouling of nanofiltration membranes by dyes during brine recovery from textile dye bath wastewater. Chem. Eng. J..

[34] Sun S.-Y., Cai L.-J., Nie X.-Y., Song X., Yu J.-G. (2015). Separation of magnesium and lithium from brine using a Desal nanofiltration membrane. J. Water Process. Eng..

[35] González A.P., Ibáñez R., Gómez P., Urtiaga A., Ortiz I., Irabien J. (2015). Nanofiltration separation of polyvalent and monovalent anions in desalination brines. J. Membr. Sci..

[36] Hilal N., Al-Zoubi H., Darwish N.A., Mohammad A.W. (2007). Performance of Nanofiltration Membranes in the Treatment of Synthetic and Real Seawater. Sep. Sci. Technol..

[37] Rainwater F.H., Thatcher L.L. (1960). Methods for Collection and Analysis of Water Samples.

[38] Fishman M.J., Friedman L.C. (1989). Methods for Determination of Inorganic Substances in Water and Fluvial Sediments.

[39] American Society for Testing and Materials (2002). ASTM C469/Standard Test Method for Static Modulus of Elasticity and Poisson’s Ratio of Concrete in Compression.

[40] ASTM International (2004). Annual Book of ASTM Standards.

[41] Freeze R., Cherry J. (1979). Groundwater.

[42] Mohallel S. (2009). Hydrochemistry and Treatment of Groundwater in the Area between Mersa Matruh and El Salloum, Egypt. Master’s Thesis.

[43] Langelier W.F. (1936). The Analytical Control of Anti-Corrosion Water Treatment. J. Am. Water Works Assoc..

[44] Langelier W.F. (1946). Chemical Equilibria in Water Treatment. J. Am. Water Works Assoc..

[45] Collins A. (1975). Geochemistry of Oilfield Waters.

[46] Mix A.E. (1944). Report on Waters, Brine and Salt. J. Assoc. Anal. Chem..

[47] Jiang S., Li Y., Ladewig B.P. (2017). A review of reverse osmosis membrane fouling and control strategies. Sci. Total Environ..

[48] Schaep J., Vandecasteele C., Mohammad A.W., Bowen W.R. (2001). Modelling the retention of ionic components for different nanofiltration membranes. Sep. Purif. Technol..

[49] Fritzmann C., Löwenberg J., Wintgens T., Melin T. (2007). State-of-the-art of reverse osmosis desalination. Desalination.

[50] Eriksson P., Kyburz M., Pergande W. (2005). NF membrane characteristics and evaluation for sea water processing applications. Desalination.

[51] Park M., Park J., Lee E., Khim J., Cho J. (2015). Application of nanofiltration pretreatment to remove divalent ions for economical seawater reverse osmosis desalination. Desalin. Water Treat..

[52] Hilal N., Al-Zoubi H., Mohammad A., Darwish N. (2005). Nanofiltration of highly concentrated salt solutions up to seawater salinity. Desalination.

[53] Mänttäri M., Pekuri T., Nyström M. (2004). NF270, a new membrane having promising characteristics and being suitable for treatment of dilute effluents from the paper industry. J. Membr. Sci..

[54] Bowen W., Welfoot J.S. (2002). Modelling the performance of membrane nanofiltration-critical assessment and model development. Chem. Eng. Sci..

[55] El-Manharawy S., Hafez A. (2003). A new chemical classification system of natural waters for desalination and other industrial uses. Desalination.

[56] Schaep J., Vandecasteele C. (2001). Evaluating the charge of nanofiltration membranes. J. Membr. Sci..

[57] Schaep J. (2001). Nanofiltration for the Removal of Ionic Components from Water. Ph.D. Thesis.

[58] Ahmad A., Ooi B., Choudhury J. (2003). Preparation and characterization of co-polyamide thin film composite membrane from piperazine and 3,5-diaminobenzoic acid. Desalination.

[59] Stiff H.A., Davis L.E. (1952). A method for predicting the tendency of oil field waters to deposit calcium carbonate. J. Pet. Technol..

[60] Telzhensky M., Birnhack L., Lehmann O., Windler E., Lahav O. (2011). Selective separation of seawater Mg^2+^ ions for use in downstream water treatment processes. Chem. Eng. J..

[61] Hussain A., Abashar M., Al-Mutaz I. (2007). Influence of ion size on the prediction of nanofiltration membrane systems. Desalination.

[62] Antropov L.I., Anissimov A. (1979). Electrochimie Théorique.

